# Candida auris Screening of High-Risk Patients: A Descriptive Comparison of 2 Strategies.

**DOI:** 10.1017/ash.2024.322

**Published:** 2024-09-16

**Authors:** Laura Pedersen, Aldo Barajas-Ochoa, Kaila Cooper, Jenna Price, Kathryn Hannum, Yvette Major, Patrick R Ching, Barry Rittmann, Michelle Doll

**Affiliations:** VCU Health; Virginia Commonwealth University; Nursing VCU Health; VCU Health Healthcare Infection Prevention Program; VCU Health System

## Abstract

**Background:** Candida auris infection is associated with high morbidity and mortality. C. auris can persist in the healthcare environment and is associated with outbreaks. We compare screening strategies for C. auris in two high-risk patient populations. **Methods:** Our center is a tertiary, 865-bed hospital. In the context of known regional outbreaks of C. auris in post-acute care (PAC) facilities, we experienced extended clusters of apparent C. auris acquisition across several hospital units. Hospital acquisition was defined as new C. auris in clinical cultures in patients with no known history of C. auris colonization/infection. We performed point prevalence surveys (PPS) on affected units weekly until all tests were negative for two consecutive weeks. We also initiated admission screening for C. auris for patients admitted from PAC. All screening swabs were collected per CDC’s procedure. Tests were performed either by RT-PCR or Chromagar C. auris media, depending on availability. We compared the overall positivity rates of exposure PPS versus PAC admission screenings using Z-test for two proportions with statistical significance set at p < 0 .05 **Results:** From 2/2023-12/2023, a total of 533 tests on 367 unique patients were processed during PPS; 512 tests were negative and 21 were positive (3.9% positivity rate). Three additional samples were either unable to be processed or indeterminate. There were 68 patients who had repeat testing weekly for ≥2 weeks. Most remained negative, but 5 tested positive after variable amounts of negative-week intervals: 3 patients at week 2, 1 patient at week 4 and 1 patient at week 5. From 8/2023 to 12/2023, a total of 89 patients admitted from 35 different PAC facilities underwent admission screening for C. auris. Only three patients were positive (3.4%), each from a different facility. The difference in the positivity rates between PPS and PAC was not statistically significant (Z-score 0.25, p = 0.79). **Discussion:** Our C. auris screening strategies found similar positivity rates for patients admitted to the hospital from PACs compared to targeted PPS in the setting of apparent hospital acquisition events. These strategies may be considered as complementary. Facilities experiencing apparent acquisition events should consider screening high-risk admissions to identify and isolate colonized patients, particularly if standard infection prevention practices are being performed with high fidelity.

